# Pesticin-Like Effector VgrG3^cp^ Targeting Peptidoglycan Delivered by the Type VI Secretion System Contributes to Vibrio cholerae Interbacterial Competition

**DOI:** 10.1128/spectrum.04267-22

**Published:** 2023-01-10

**Authors:** Ming Liu, Meng-Yu Zhao, Heng Wang, Zeng-Hang Wang, Zhao Wang, Ying Liu, Yin-Peng Li, Tao Dong, Yang Fu

**Affiliations:** a School of Medicine, Southern University of Science and Technology, Shenzhen, China; b The First Affiliated Hospital, Southern University of Science and Technology, Shenzhen, China; c State Key Laboratory of Microbial Metabolism, Joint International Research Laboratory of Metabolic & Developmental Sciences, School of Life Sciences and Biotechnology, Shanghai Jiao Tong University, Shanghai, China; d Department of Immunology and Microbiology, School of Life Sciences, Southern University of Science and Technology, Shenzhen, China; South China Sea Institute of Oceanology

**Keywords:** T6SS, evolved VgrG, interspecies interactions, *Vibrio cholerae*, peptidoglycan

## Abstract

Vibrio cholerae can utilize a type VI secretion system (T6SS) to increase its intra- and interspecies competition. However, much still remains to be understood about the underlying mechanism of this intraspecies competition. In this study, we isolated an environmental V. cholerae strain E1 that lacked the typical virulence factors toxin-coregulated pilus and cholera toxin and that encoded a functional T6SS. We identified an evolved VgrG3 variant with a predicted C-terminal pesticin-like domain in V. cholerae E1, designated VgrG3^cp^. Using heterologous expression, protein secretion, and peptidoglycan-degrading assays, we demonstrated that VgrG3^cp^ is a T6SS-dependent effector harboring cell wall muramidase activity and that its toxicity can be neutralized by cognate immunity protein TsiV3^cp^. Site-directed mutagenesis proved that the aspartic acid residue at position 867 is crucial for VgrG3^cp^-mediated antibacterial activity. Bioinformatic analysis showed that genes encoding VgrG3^cp^-like homologs are distributed in *Vibrio* species, are linked with T6SS structural genes and auxiliary genes, and the *vgrG3^cp^-tsiV3^cp^* gene pair of V. cholerae probably evolved from Vibrio anguillarum and Vibrio fluvialis via homologous recombination. Through a time-lapse microscopy assay, we directly determined that cells accumulating VgrG3^cp^ disrupted bacterial division, while the cells continued to increase in size until the loss of membrane potential and cell wall breakage and finally burst. The results of the competitive killing assay showed that VgrG3^cp^ contributes to V. cholerae interspecies competition. Collectively, our study revealed a novel T6SS E-I pair representing a new T6SS toxin family which allows V. cholerae to gain dominance within polymicrobial communities by T6SS.

**IMPORTANCE** The type VI secretion system used by a broad range of Gram-negative bacteria delivers toxic proteins to target adjacent eukaryotic and prokaryotic cells. Diversification of effector proteins determines the complex bacterium-bacterium interactions and impacts the health of hosts and environmental ecosystems in which bacteria reside. This work uncovered an evolved valine-glycine repeat protein G3, carrying a C-terminal pesticin-like domain (VgrG3^cp^), which has been suggested to harbor cell wall hydrolase activity and is able to affect cell division and the integrity of cell wall structure. Pesticin-like homologs constitute a family of T6SS-associated effectors targeting bacterial peptidoglycan which are distributed in *Vibrio* species, and genetic loci of them are linked with T6SS structural genes and auxiliary genes. T6SS-delivered VgrG3^cp^ mediated broad-spectrum antibacterial activity for several microorganisms tested, indicating that VgrG3^cp^-mediated antimicrobial activity is capable of conferring bacteria a competitive advantage over competitors in the same niches.

## INTRODUCTION

The Gram-negative bacterium Vibrio cholerae is the causative agent of the disease cholera (1). During colonization, V. cholerae not only needs to face pressures such as low pH, hypoxia, and the intestinal flora in host (2), but also needs to evade the natural aquatic environment’s phage lysis, eukaryotic predation, and bacterial competition in a common niche (3). Type VI secretion systems (T6SSs) are widely distributed in ~25% of Gram-negative bacteria and can kill neighbor cells by delivering toxic effectors in a contact-dependent manner, while sister cells with the cognate immunity proteins can survive (4–6). Therefore, T6SS is an important weapon for bacteria to dominate their niche and to mediate host pathogenicity (7–13). T6SS consists of a transmembrane complex, baseplate (14, 15), Hcp inner tube coated with a VipA/B sheath (16), and a VgrG trimer complex sharpened by PAAR (proline-alanine-alanine-arginine repeat) proteins (17–19).

A broad diversity of antibacterial toxins delivered by T6SS has been described (20, 21). Depending on the action sites of effectors, they can roughly be divided into two categories: (i) toxins targeting the cell envelope, including membrane lipase effectors TseL (22) and PldA (23), a pore-forming VasX (24), and cell wall hydrolases VgrG3 (22, 25) and Tge (26); (ii) toxins targeting the cell cytoplasm, including proteases EvpP (27) and TecA (28) and nucleosidase Rhs (29) and Tce1 (30). Cell wall peptidoglycan (PG) is a potentially fatal target of attack, because it is important for maintaining the cell shape and resisting mechanical and osmotic damage. T6SS effectors targeting peptidoglycan are further divided into two categories: one acts as an amidase, cleaving within peptide stems or cross-links (31), and the other acts as a glycoside hydrolase that cleaves the glycan backbone (26).

T6SS as a macromolecular machine delivers effectors in two ways: (i) a toxic domain fused within the structural component, called specialized effectors; and (ii) noncovalent interaction with structural components called cargo effectors (32). Among the reported effectors, the first described specialized effector was VgrG1 of V. cholerae, whose C-terminal extension contains an actin–cross-linking domain (ACD) (19). Since the discovery of VgrG1, several types of specialized effector have been characterized, including VgrG3, PAAR, and Hcp-ET (22, 33, 34). In contrast, cargo effectors are independent toxin units whose translocations require interacting directly with the proteins encoded by the adjacent structural genes, such as TseH and PAAR2 of V. cholerae (35), Tse2 and Hcp1 of Pseudomonas aeruginosa (36), and Tse5 and -6 and VgrG of P. aeruginosa (37).

All sequenced V. cholerae strains contain a T6SS (38, 39). Toxigenic strains commonly encode the conserved effectors, which have been studied in detail, while nontoxigenic strains harbor diverse effectors which are rarely investigated about their function (9, 38, 40, 41). In this study, we isolated an environmental strain, E1, with a functional T6SS. The T6SS of E1 consists of a large cluster, an Aux1 cluster and Aux2 cluster, encoding a C-terminal extension of the VgrG effector (named VgrG3^cp^), two effectors of VgrG1 lacking an ACD, and a potential lipase effector (named Tle1) and a known VasX effector, respectively. Here, we characterized VgrG3^cp^, which has a pesticin-like domain that differs from the C terminus of VgrG3 and a lysozyme-like domain encoded by toxigenic strains (25). T6SS-dependent VgrG3^cp^ is a periplasm-acting toxin, and its C-terminal extension domain possesses cell wall hydrolase activity that inhibits cell division and breaks the integrity of the cell wall, resulting in intoxication of target cells. Moreover, intoxicated cells can be rescued by expressing the cognate immunity protein TsiV3^cp^. VgrG3^cp^-like homologs constitute a family of T6SS-associated effectors that target bacterial peptidoglycan and are distributed in V. cholerae, Vibrio anguillarum, and Vibrio fluvialis and are often linked with T6SS structural genes and auxiliary genes. Competitive killing assays showed that T6SS or its secreted VgrG3^cp^ enables V. cholerae to dominate the competition among multiple bacterial communities. Taken together, our findings showed that VgrG3^cp^-mediated antibacterial activity confers environmental strain E1 competition advantages in polymicrobial environments.

## RESULTS

### The C-terminal domain of VgrG3^cp^ confers antibacterial activity.

**FIG 1:**
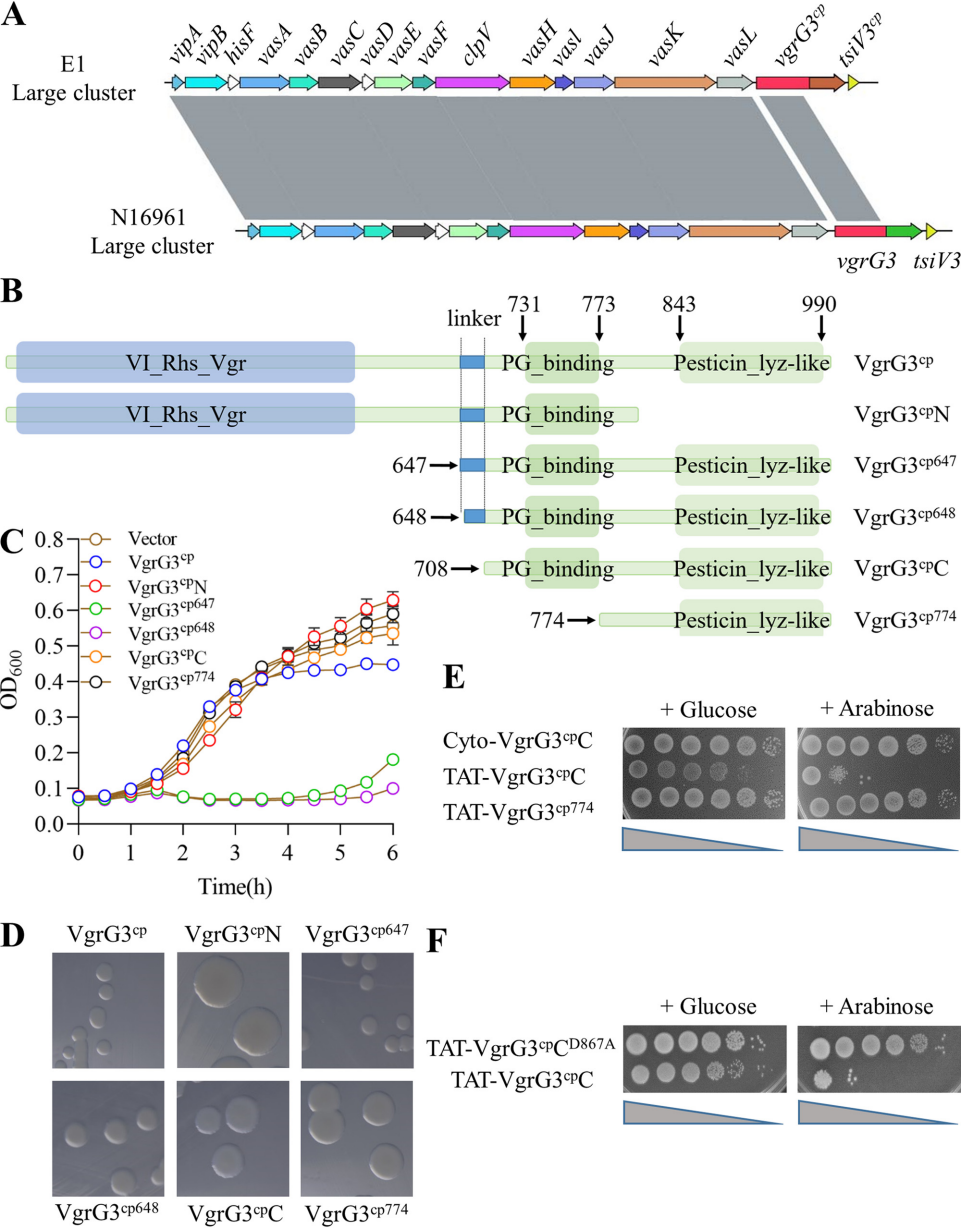
Identification of VgrG3^cp^ bactericidal activity. (A) The homology comparison of large cluster genes between V. cholerae N16961 and E1. (B) Schematic view of VgrG3^cp^ truncation expression assays. Truncated mutants included VgrG3^cp^N, which retained the *N*-terminal structure, or a series of C-terminal extended domains (VgrG3^cp647^, VgrG3^cp648^, VgrG3^cp^C [708 to the end], and VgrG3^cp774^). (C) Identification of VgrG3^cp^ toxic region. E. coli cells expressing VgrG3^cp^ full-length and truncated variants were cultured in LB medium supplemented with 50 μM IPTG, and the OD_600_ was measured at the time points indicated. The experiments were run at least three times. (D) Effects of VgrG3^cp^ and its variants on colony morphology. E. coli cells bearing proteins indicated were spread on LB plates with corresponding antibiotic; a single colony was imaged after 36 h. All images were gained at the same magnification. (E and F) Toxicity of VgrG3^cp^ variants in periplasmic expression. E. coli BL21 cells expressing cytoplasmic VgrG3^cp^C (Cyto-VgrG3^cp^C), periplasmic VgrG3^cp^C (TAT-VgrG3^cp^C), VgrG3^cp774^ (TAT-VgrG3^cp774^), and VgrG3^cp^C^D867A^ (TAT-VgrG3^cp^C^D867A^) were incubated on LB agar plates in the presence of arabinose (induction) or glucose (repression) at 37°C overnight and imaged. The representative data (D to F) from three independent biological replicates are displayed.

In this study, we isolated an environmental V. cholerae strain E1 constitutively expressing T6SS (see Fig. S1A to C in the supplemental material). Using an intraspecies killing assay, we found that strain E1 outcompeted C6706 in a T6SS-dependent manner (Fig. S1D), showing that E1 may encode new T6SS effectors and C6706 lacks genes coding the corresponding cognate immunity proteins. Through comparative bioinformatics analysis, we noted that reference strain N16961 encodes a cell wall-targeting VgrG3 with a lysozyme-like domain (25), while strain E1 carries a pesticin-like domain in the VgrG C-terminal extension designated VgrG3^cp^ (Fig. 1A and B; Fig. S2A).

Sequence analysis of VgrG3^cp^ using the PFAM database suggested that its toxic activity falls within a putative pesticin-like domain located at the C terminus (Fig. 1B). To verify this prediction, we examined the toxicity of the full-length VgrG3^cp^ and its truncated mutants lacking a TAT signal (Fig. 1B) when expressed in Escherichia coli BL21(DE3). As expected, expression of truncated VgrG3^cp647^ or VgrG3^cp648^ resulted in an obvious inhibition in E. coli growth. Interestingly, the two truncated VgrG3^cp^ forms (VgrG3^cp647^ and VgrG3^cp648^) were even more harmful than the full-length VgrG3^cp^ (Fig. 1C), which might have been because the full-length VgrG3^cp^ could form a homologous trimer and decreased the translocation efficiency into the periplasm of the target cell. In contrast, cells expressing a conserved *N* terminus VgrG3^cp^N and truncated variants VgrG3^cp^C (i.e., residues 708 to the end) and VgrG3^cp774^ grew well, similar to the empty vector control. Furthermore, the intoxicated cells also displayed smaller colonies (Fig. 1D). These results demonstrated that the C-terminal region of VgrG3^cp^ (647 to the end) plays a vital role for antibacterial activity.

Ho et al. suggested that there is a linker site within the VgrG3 protein as well as in VgrG3^cp^ (Fig. S2A) which acts as a signal peptide directing translocation of the C-terminal toxic extension domain (22). Therefore, we investigated whether the linker region has a role in the transfer of VgrG3^cp^C. The *N* terminal of VgrG3^cp^C was fused with a twin arginine translocation signal sequence (TAT) and then cloned into an arabinose-inducible vector, pBAD24, to produce periplasmic protein TAT-VgrG3^cp^C. Under the arabinose-inducible conditions, surviving BL21 cells expressing TAT-VgrG3^cp^C were significantly reduced compared to cells expressing Cyto-VgrG3^cp^C (Fig. 1E). Addition of glucose to repress the arabinose-inducible promoter rescued cells expressing TAT-VgrG3^cp^C (Fig. 1E), showing that VgrG3^cp^C still retained enzyme activity but was not transferred to the periplasm of the target cell. Combined with the results in Fig. 1C and D, these findings strongly suggested that a linker region indeed functions in translocation. In addition, we tested the effect of a PG-binding site (VgrG3^cp774^) on VgrG3^cp^-mediated antibacterial activity. Cells expressing TAT-VgrG3^cp774^ failed to induce growth inhibition in the presence of arabinose, compared to cells expressing TAT-VgrG3^cp^C (Fig. 1E). These results indicated that the linker sequence and PG-binding site coupled with a pesticin-like domain are important for VgrG3^cp^-induced toxicity.

### The D867 site is vital to VgrG3^cp^-catalyzed activity.

VgrG3^cp^C shares conserved catalytic site with pesticin at the amino acid level (Fig. S2B). Three residues of pesticin (glutamic acid at residue 178 [E178], T201, and D207) have been shown to be important for catalysis (42); two of the residues (T201 and D207) are conserved between pesticin and the VgrG3^cp^ effector. To search for a potential active site for VgrG3^cp^, we mutated D867 aspartic acid residues to alanines (corresponding to D207 in pesticin) and tested the toxicity for periplasmic expression of VgrG3^cp^C and its mutant in E. coli BL21(DE3). Expression of TAT-VgrG3^cp^C resulted in reduced survival, while expression of a TAT-VgrG3^cp^C^D867A^ mutant did not (Fig. 1F), indicating that the D867 site is essential to VgrG3^cp^-catalyzed antibacterial activity.

### VgrG3^cp^ is a peptidoglycan-targeting toxin that dysregulates cell division and membrane potential.

**FIG 2:**
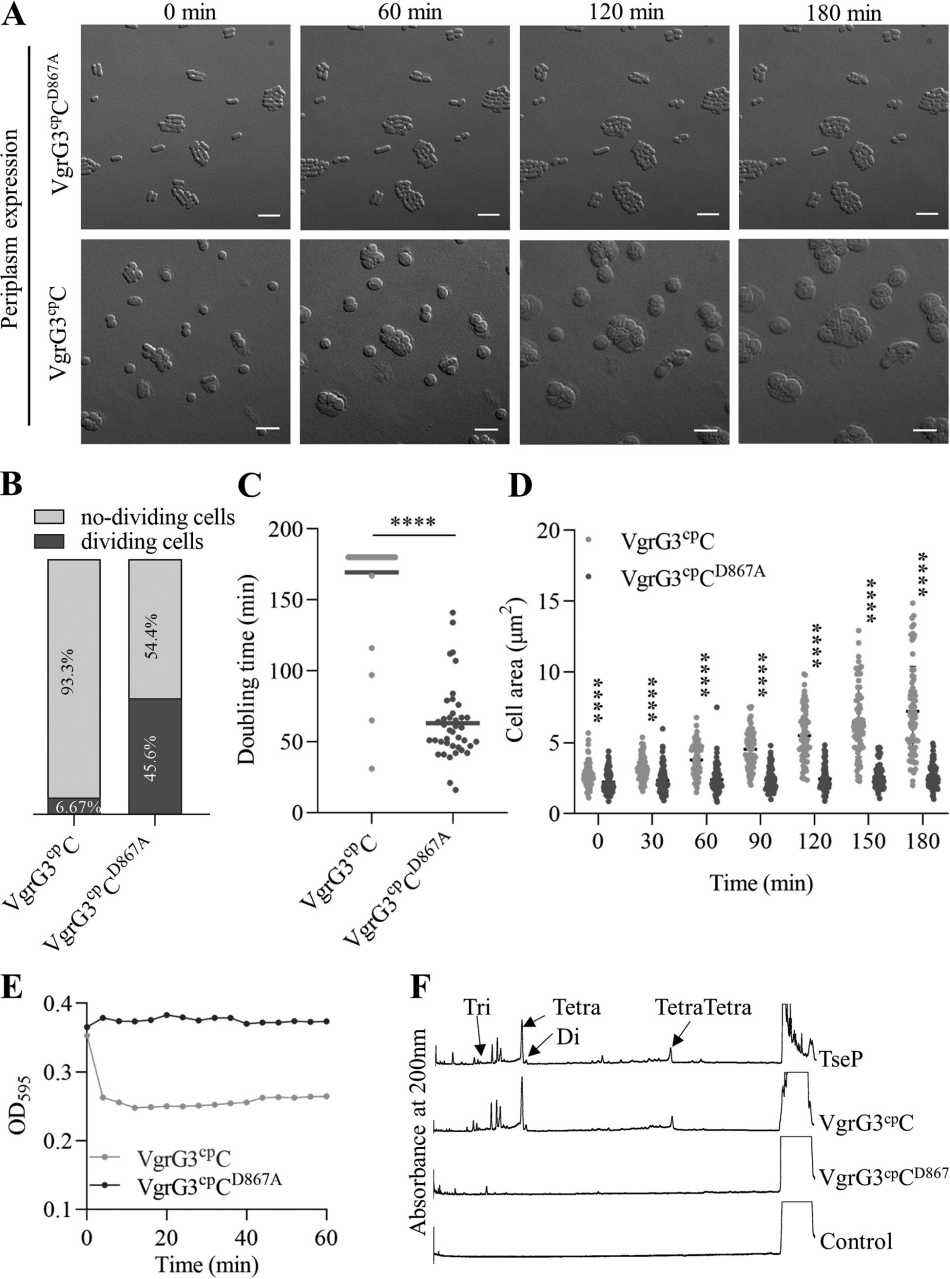
VgrG3^cp^ is a periplasmic-targeting toxin that dysregulates cell division and degrades the peptidoglycan. (A) Time-lapse microscopy of E. coli cells expressing TAT-VgrG3^cp^C or TAT-VgrG3^cp^C^D867A^ grown on LB agar pads containing 0.2% l-arabinose (induced). Scale bar, 5 μm. Time stamps units are minutes. A representative image from three independent biological replicates is shown. (B) Percentages of dividing and nondividing cells observed in panel A between 0 and 3 h. Approximately 90 cells were calculated under each condition. (C) Doubling time (in minutes) of cells that divided and are quantified in panel B. Error bars represent the means ± standard deviations (SD) of ~40 cells under each condition. Significance was calculated using an unpaired Student's *t* test. ****, *P < *0.0001. (D) Colony surface areas of cells observed in panel A between 0 and 3 h. The colony areas were measured by ImageJ software. Error bars represent the means ± SD of ~90 cells at each time point. Significance was calculated using an unpaired Student's *t* test. ****, *P < *0.0001. (E) Peptidoglycan-degrading assays. Purified E. coli cell wall was treated with VgrG3^cp^C and its catalytic mutant, VgrG3^cp^C^D867A^, respectively. Digestion of the insoluble cell wall resulted in reduction of the OD_595_. The experiments were run two times, and representative data are shown. Purified proteins were analyzed by SDS-PAGE (see Fig. S3A in the supplemental material). (F) UPLC chromatograms of E. coli peptidoglycan products digested by purified VgrG3^cp^C and VgrG3^cp^C^D867A^. Peaks indicated are based on mass spectrometry. Mass spectrometry data are shown in Fig. S3C to F.

In order to gain further mechanism insights into VgrG3^cp^-induced toxicity, we performed time-lapse microscopy to observe the growth and morphology of single E. coli BL21 cells carrying pBAD24-TAT-VgrG3^cp^C or pBAD24-TAT-VgrG3^cp^C^D867A^. In the presence of 0.2% l-arabinose and voltage-dependent dye DiBAC4(3), which upon cell depolarization can enter cells and stain cellular membranes (43), E. coli BL21 cells expressing TAT-VgrG3^cp^C^D867A^ grew well over a time frame of 3 h (Fig. 2A to D; Movie S1). However, intoxicated cells expressing TAT-VgrG3^cp^C displayed a series of changes in cell division and morphology (Fig. 2A to D; Movie S2). At starting time points (within 1 h), we captured that cells producing VgrG3^cp^C underwent a shape change from rod-shaped to spherical cells (Movie S3, yellow arrow) as well as from spherical cells to burst cells (Movie S3, green arrow), while cells expressing VgrG3^cp^C^D867A^ maintained normal growth and shape (Movie S1). Between 0 and 3 h of incubation in the presence of arabinose, only 6.67% of intoxicated cells were observed to undergo 1 round of cell division, with a doubling time of ~167 min, while 45.6% of cells expressing TAT-VgrG3^cp^C^D867A^ divided in at least 1 round in the same time scale, with a doubling time of ~64 min (Fig. 2B and C), indicating that VgrG3^cp^C-mediated toxicity inhibits cell division or increases doubling times. Although VgrG3^cp^C inhibited cell division, the cell size was increasing. After inducing for 3 h, intoxicated cells had an average colony surface area of ~7 μm^2^, and a subset of cells (17 of 90 intoxicated cells, 18.9%) stained green and then burst (Fig. 2D; Movies S2 and S3), suggesting the intoxicated cells lost membrane potential and released intracellular content, while cells expressing TAT-VgrG3^cp^C^D867A^ displayed an average colony surface area of ~2 μm^2^ and maintained the normal membrane potential (Fig. 2D; Movie S1).

According to the results indicating that VgrG3^cp^C contains PG binding site and a pesticin-like domain and mediates the formation of spherical cells, we speculated that VgrG3^cp^ may target peptidoglycan as a potential substrate. To explore this possibility, we purified glutathione *S*-transferase (GST)-tagged VgrG3^cp^C and its catalytic VgrG3^cp^C^D867A^ mutant using the AKTA Explorer protein purified system (Fig. S3A) and incubated them with purified E. coli MG1655 cell wall sacculi (44). The change of optical density (OD) of mixtures was monitored at 600 nm as digestion of the insoluble cell wall results in reduction of the OD_600_. Through peptidoglycan-degrading assays, wild-type VgrG3^cp^C reduced the optical density of cell walls within 5 min to 30 min, while VgrG3^cp^C^D867A^ had no effect on optical density of cell walls (Fig. 2E), suggesting that VgrG3^cp^ has cell wall hydrolase activity.

Next, in order to explore whether VgrG3^cp^ cleaves the backbone or cross-chain of peptidoglycan, we analyzed the soluble contents of cell walls treated with VgrG3^cp^C or VgrG3^cp^C^D867A^ by using ultraperformance liquid chromatography-quadrupole time of flight mass spectrometry (UPLC-QTOF-MS). Like known peptidoglycan-degrading effector TseP (45), samples treated with VgrG3^cp^C could readily detect *N*-acetylmuramic acid-*N*-acetylglucosamine (NAM-NAG) with various lengths of peptides, whereas no peptides were captured in samples treated with VgrG3^cp^C^D867A^ (Fig. 2F; Fig. S3C to F), further supporting that pesticin-like VgrG3^cp^ catalyzes the cleavage of peptidoglycan chains.

### VgrG3^cp^-TsiV3^cp^ consists of an antibacterial effector-immunity protein pair.

**FIG 3:**
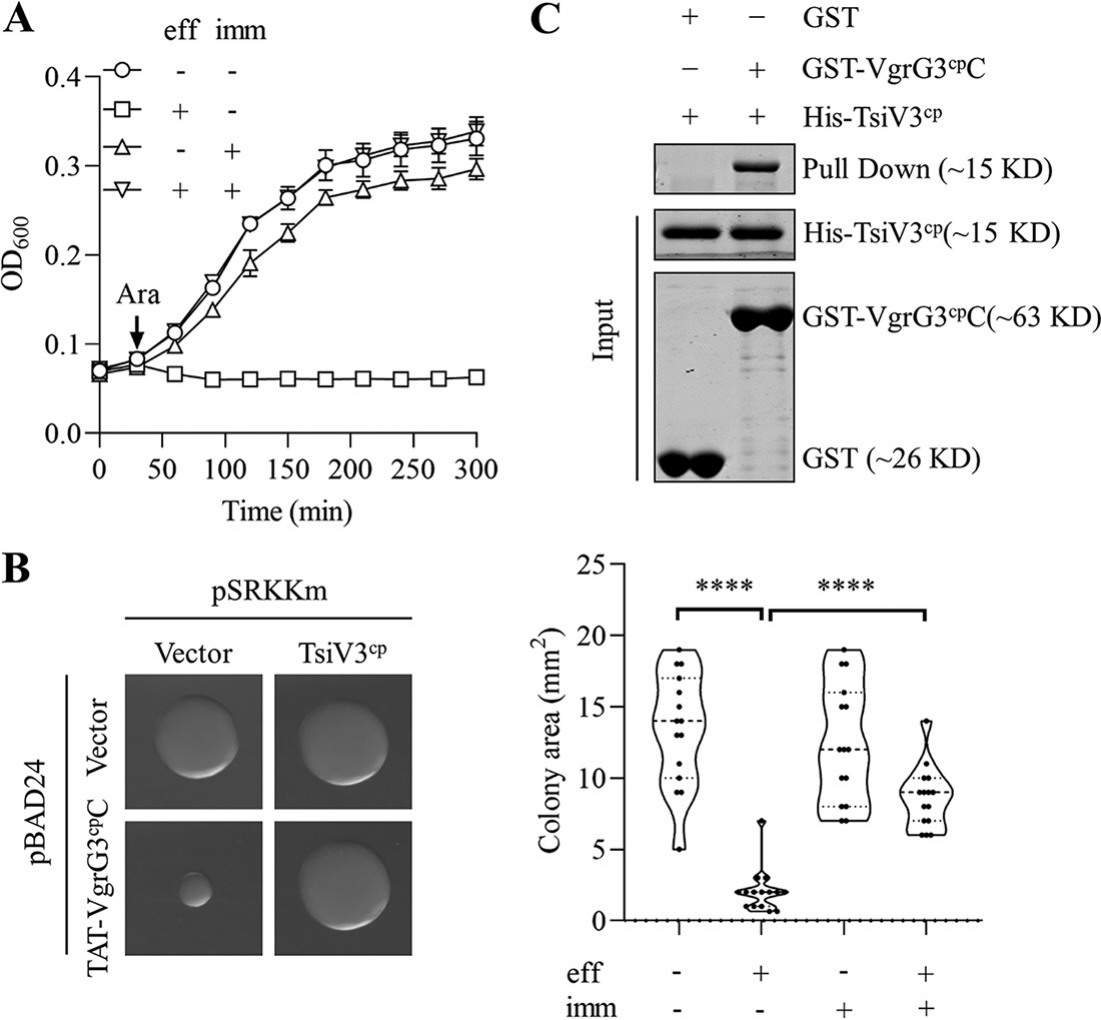
TsiV3^cp^ is the cognate immunity protein to VgrG3^cp^. (A) E. coli BL21 cells expressing TAT-VgrG3^cp^C (effector) and TsiV3^cp^ (immunity) were cultured in LB medium with 50 μM IPTG (for pSRKKm) and 0.2% l-arabinose (for pBAD24) at 37°C; the OD_600_ was monitored at the indicated time points. (B) Quantification of a single colony area. E. coli cells in panel A were spread onto LB agar plates with 0.03 mM IPTG and grown at 37°C. All representative images were gained at the same magnification (left). The areas of 15 single colonies per group were measured for comparison (right). Significance was calculated using an unpaired Student's *t* test. ****, *P < *0.0001. (C) *In vitro* pulldown assay of TsiV3^cp^ and VgrG3^cp^C. The purified GST-VgrG3^cp^C and His-TsiV3^cp^ were mixed and incubated with MagneGST glutathione beads, and bound proteins were eluted and detected by SDS-PAGE. Data in panels B and C are representative of three independent replicates.

Each T6SS effector gene and its cognate immunity protein gene are genetically linked (46). Immunity proteins can neutralize the toxicity of cognate effectors to prevent self-intoxication or sibling intoxication (4). Using coexpression assays, we examined the effect of the *vgrG3^cp^* downstream gene (named *tsiV3^cp^*) on VgrG3^cp^ toxicity. The growth rates of BL21 cells expressing TsiV3^cp^ and either VgrG3^cp^C or TAT-VgrG3^cp^C were measured. Results showed that growth of BL21 cells was completely abolished when only VgrG3^cp^C was expressed in periplasm, while the growth inhibition was relieved when coexpressed with TsiV3^cp^ (Fig. 3A). Meanwhile, the TAT-VgrG3^cp^C-induced smaller colonies could be restored to normal size by expression of TsiV3^cp^ (Fig. 3B). To further verify that TsiV3^cp^ can interact with VgrG3^cp^C, the truncated VgrG3^cp^C with a GST tag and TsiV3^cp^ with a His tag were purified (Fig. S3A and B). In a GST pulldown assay, we observed that TsiV3^cp^ could be captured by GST-VgrG3^cp^C but not GST protein, suggesting that TsiV3^cp^ could detoxify VgrG3^cp^C by direct interaction (Fig. 3C). These data confirmed that TsiV3^cp^ and VgrG3^cp^ are an effector-immunity protein pair.

### Secretion of VgrG3^cp^ requires an active T6SS.

**FIG 4:**
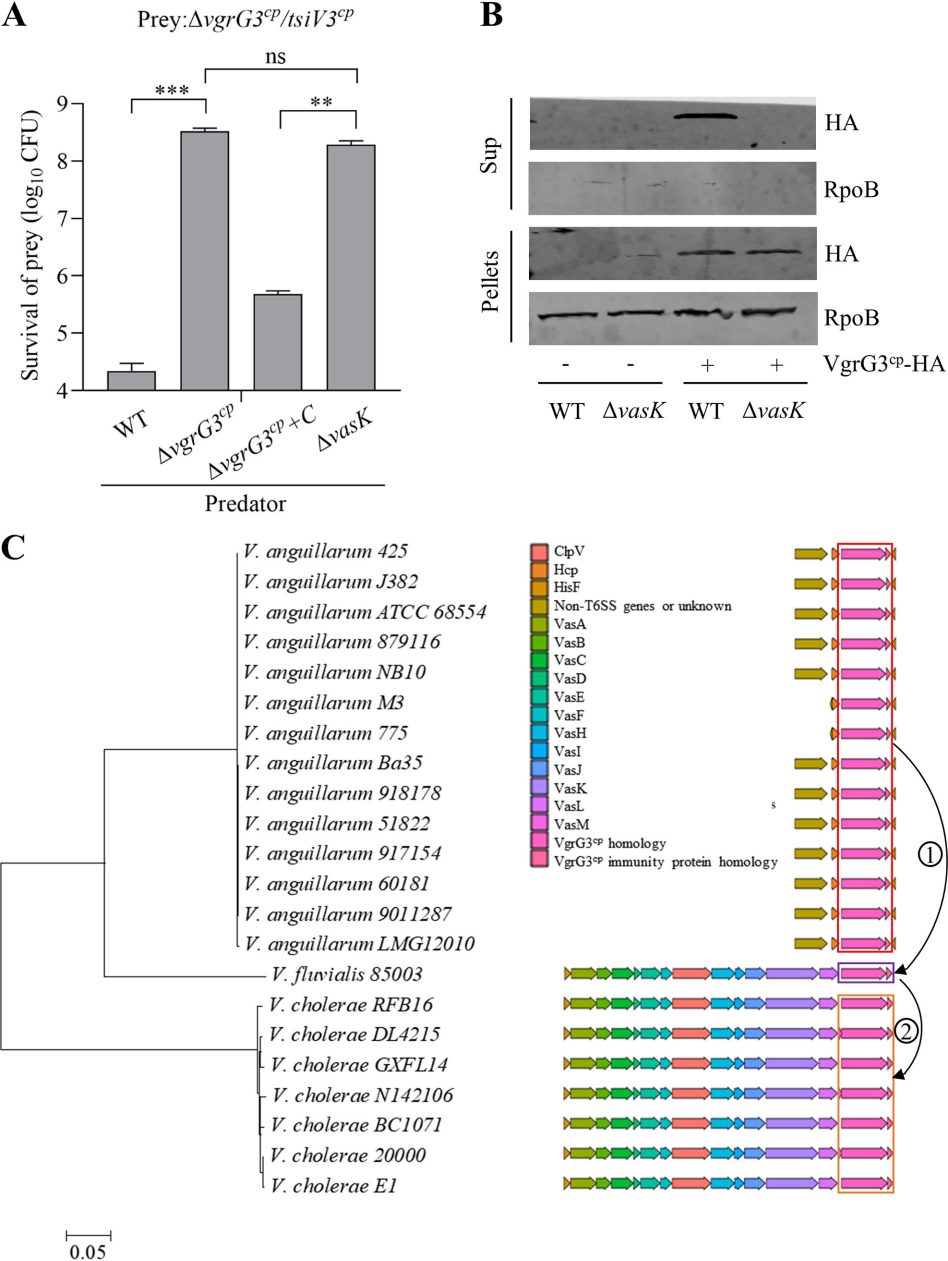
Secretion of VgrG3^cp^ depends on a T6SS. (A) Quantification of prey strain after coincubation with the indicated predator strains. The Δ*vgrG3^cp^/tsiV3^cp^* mutant as prey was incubated with predator strains including V. cholerae E1 WT, Δ*vasK*, Δ*vgrG3^cp^/tsiV3^cp^*, and Δ*vgrG3^cp^* complemented with *vgrG3^cp^* (Δ*vgrG3^cp^+C*) for 4 h. Surviving prey cells were serially diluted and determined on the LB plate containing streptomycin. Bars represent the means ± SD from three independent experiments. Significance was calculated using an unpaired Student's *t* test. ns, nonsignificant; **, *P* < 0.01; ***, *P* < 0.001. (B) Effect of T6SS on secretion of VgrG3^cp^. Whole cells and supernatants of V. cholerae E1 WT and ΔvasK mutant expressing either pSRKKm vector (−) or VgrG3^cp^ fused with HA tag (+) were analyzed by Western blotting. RNA polymerase beta subunit (RpoB) was used as an internal reference. Representative data from three independent replicates are displayed. (C) Maximum-likelihood phylogeny of strains containing VgrG3^cp^ homologs. Sequences were aligned and maximum-likelihood phylogeny was constructed with a bootstrap of 1,000. The corresponding species and operon structures are displayed; colors indicate the conserved T6SS proteins. The arrows represent that V. cholerae acquired the potential evolutionary routes of *vgrG3^cp^-tsiV3^cp^* EI pair genes.

The evolved effectors VgrG1 and VgrG3 require a functional T6SS for delivery (19, 25). To identify the role of functional T6SS for VgrG3^cp^-mediated toxicity, we constructed a *vgrG3^cp^* deletion (Δ*vgrG3^cp^*), a complementation of VgrG3^cp^ (Δ*vgrG3^cp^+C*), and an inactive T6SS mutant (Δ*vasK*) as attackers and a double mutant lacking *vgrG3^cp^* and *tsiV3^cp^* genes (Δ*vgrG3^cp^/tsiV3^cp^*) as prey. Using competitive killing assays, we found that the wild-type (WT) strain was able to significantly reduce the number of survival prey compared to the Δ*vasK* mutant, while the Δ*vgrG3^cp^* strain abolished killing of target cells and could be recovered by complementing with VgrG3^cp^, suggesting a functional T6SS is required to VgrG3^cp^-mediated specific killing (Fig. 4A). To further confirm this, we fused VgrG3^cp^ with an hemagglutinin (HA) tag and expressed HA-VgrG3^cp^ in V. cholerae E1 WT and Δ*vasK*. Western blot analysis showed that VgrG3^cp^ protein could be detected in the supernatant of the wild-type strain but not in the Δ*vasK* mutant. Meanwhile, VgrG3^cp^ was detected in cells of both strains overexpressing VgrG3^cp^ (Fig. 4B). The results confirmed that the secretion of VgrG3^cp^ depends on a functional T6SS.

### VgrG3^cp^ homologs are distributed in *Vibrio* bacteria.

We next investigated the distribution of VgrG3^cp^ homologs in bacteria by BLAST analysis and sorted out 23 VgrG3^cp^ homologs in V. cholerae, *V. fluvialis* and V. anguillarum. By analyzing the results of genomic context of VgrG3^cp^, we found that VgrG3^cp^ homologs were encoded within a T6SS core cluster in *V. fluvialis* and V. cholerae or an auxiliary cluster associated with Hcp protein in V. anguillarum (Fig. 4C), suggesting that VgrG3^cp^ is a T6SS-dependent effector in *Vibrio*. There is consistently a TsiV3^cp^ immunity protein homolog downstream of VgrG3^cp^ homolog, indicating that they may play an important role in immunity against effectors in different bacteria.

### T6SS-mediated VgrG3^cp^ confers interbacterial antagonism.

**FIG 5:**
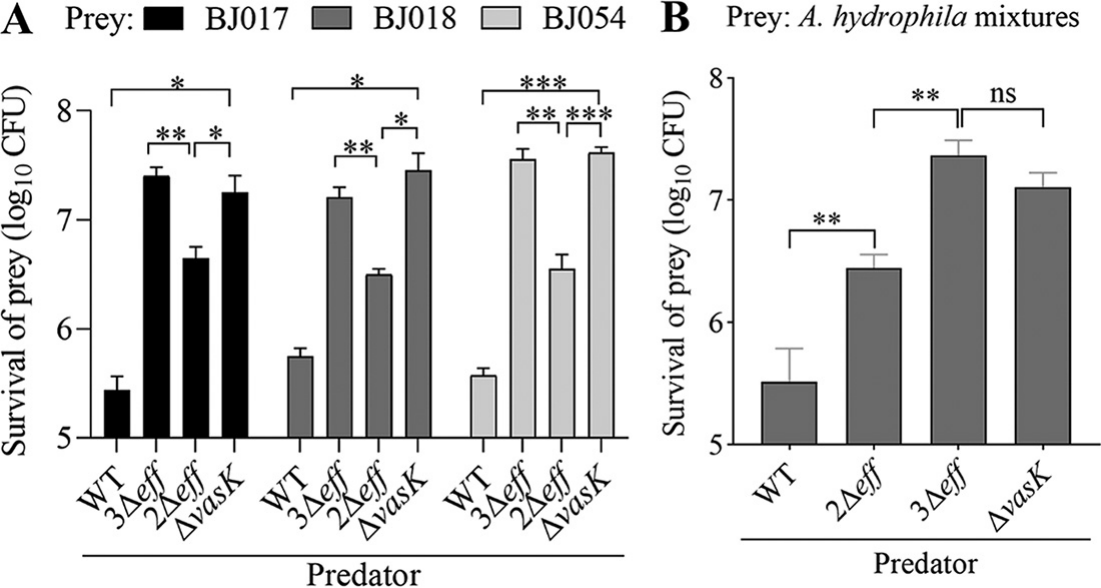
VgrG3^cp^-mediated interbacterial competition assays. Wild type, Δ*vasK*, 2Δ*eff*, and 3Δ*eff* (2Δ*eff* contains deletions of *tle1* and inactivated effector *vasX*^Δ^*^C16^*; 3Δ*eff* contains deletions of *tle1* and *vgrG3^cp^* and inactivated *vasX*^Δ^*^C16^*) as predator strains were coincubated with each of A. hydrophila prey strains BJ014, BJ017, and BJ054 individually (A) or as a mixture (B), at a ratio of 5:1 (predator:prey). After incubating for 4 h, surviving prey cells were enumerated by serial dilution onto ampicillin plates. Bars represent the means ± SD from three independent experiments. Significance was calculated using an unpaired Student's *t* test. ns, nonsignificant; *, *P < *0.05; **, *P < *0.01; ***, *P < *0.001.

In both host and environmental settings, V. cholerae relies on T6SS to compete with other organisms for limited resources and spaces. To explore the function of T6SS encoded by E1 in interaction with neighboring species, we chose Aeromonas hydrophila, a common, Gram-negative bacteria which often shares the same niche with V. cholerae (47), as prey in interbacterial competition experiment. We evaluated the survival of three A. hydrophila strains, BJ014, BJ017, and BJ054 (48), after they were individually coincubated with either E1 wild type or Δ*vasK* as predator for 4 h. Compared with the Δ*vasK* strain, the wild type notably reduced the survival of each A. hydrophila strain (Fig. 5A). We further tested the toxicity of VgrG3^cp^ on A. hydrophila. We deleted Tle1 and the second transmembrane loop (lacking A852 to F867) of VasX (the resultant VasX^ΔC16^ mutant was inactivated but allowed its secretion [49]), or all three known effectors in V. cholerae E1 to gain a double effector deletion, 2Δ*eff* (active VgrG3^cp^), and the triple effector deletion 3Δ*eff* (inactive VgrG3^cp^) as predators, respectively. Under the same killing conditions, the numbers of survival prey were significantly lower in the 2Δ*eff* mutant than in the 3Δ*eff* mutant (Fig. 5A), suggesting VgrG3^cp^ mediated the intoxication of target cells. Using the mixtures of three A. hydrophila strains as prey, we also showed that VgrG3^cp^ allowed V. cholerae E1 to dominate in an A. hydrophila population (Fig. 5B). Collectively, these data indicated that VgrG3^cp^ enables V. cholerae to gain a competitive advantage in polymicrobial communities.

## DISCUSSION

Bacteria use multiple strategies to adapt to changes in complex environments. For example, T6SSs utilized by Gram-negative bacteria play an important role in interbacterial competition and antieukaryotic predation, whose physiological significance depends on secreted effectors. Bacterial T6SSs often carry two types of effectors, specialized effectors and cargo effectors, which can target various cell components, of which periplasmic spaces seem to be the most common target (31). Specialized effectors containing dual components with structural and effector functions have been shown in different bacteria, for example, specialized PAAR, VgrG, and Hcp (17, 19, 34). Evolved VgrGs are most common in T6SS bacteria. Only VgrG1 and VgrG3, but not VgrG2, have been identified as evolved VgrG in V. cholerae so far. Here, we applied a bioinformatics approach to identify a novel evolved effector, VgrG3^cp^, with potential peptidoglycan-degrading activity and its immunity protein, TsiV3^cp^, in environmental V. cholerae E1. VgrG3^cp^ contains the VI_Rhs_Vgr superfamily in its *N* terminus, PG binding domain in the middle, and the pesticin-like domain in the C terminus. Truncated VgrG3^cp647^ and VgrG3^cp648^ variants of VgrG3^cp^ were shown to have toxicity when periplasmically expressed in E. coli cells, which differs from that of VgrG3 (22), suggesting that the C-terminal difference of VgrG3^cp^ and VgrG3 may affect the translocation efficiency. Our findings provide direct evidence that pesticin-like VgrG3^cp^ degrades PG and that the PG-binding domain and the conserved D867 aspartic acid residue within the pesticin-like domain are critical to catalytic activity. Like VgrG1 and VgrG3, VgrG3^cp^ requires a functional T6SS for delivery. Our analysis also demonstrated that VgrG3^cp^ homologs exist in diverse *Vibrio* species and are often genetically linked to T6SS structural and auxiliary gene clusters. These data demonstrated that VgrG3^cp^, with a pesticin-like domain, represents an important superfamily of cell wall-targeting T6SS effectors.

It has been reported that T6SS-associated effector-immunity genes are frequently transferred between *Vibrio* species through homologous recombination and horizontal gene transfer (50, 51). The three-letter system was used to type the V. cholerae T6SS variants based on their effector-immunity (EI) modules, with various letters representing diverse EI gene families (39). In the case of the large cluster, multiple toxic regions were fused to the 3′ end of *vgrG3*, resulting in divergent VgrG3 variants (A to M) within the large clusters. For example, the VgrG3 of toxigenic V. cholerae was identified as A-type (39). Further analysis show that pesticin-like VgrG3^cp^ belongs to C-type variants because it is highly identical to the known C-type VgrG3 variant of V. cholerae DL4215 (Fig. 4C), which has been shown to have evolved from the variable region of ancestral *vgrG*3 via homologous exchange (51). V. cholerae E1 may have resulted from a similar evolutionary mechanism to gain the *vgrG3^cp^-tsiV3^cp^* EI pair. Interestingly, the VgrG3^cp^ homolog of *V. fluvialis* and V. anguillarum are distributed in T6SS large and auxiliary clusters, respectively. Moreover, V. anguillarum is a closer relative of *V. fluvialis* than is V. cholerae, based on the core genome phylogeny (50), suggesting that a C-type VgrG3 variant of *V. fluvialis* may have been acquired from V. anguillarum via homologous recombination, which further spread into the T6SS large cluster of V. cholerae (Fig. 4C). Consistent with our current investigation, V. anguillarum consists of a weapon reservoir of V. cholerae-associated T6SS effectors that may facilitate competition between vibrios for an environmental niche (50).

Bacterial cell division and elongation is differentially controlled by peptidoglycan synthesis (52, 53). Septal peptidoglycan-synthesizing enzymes are recruited by filaments of FtsZ and FtsA to the division site. The stability of FtsZ rings at new cell poles determines the timing of cell division (54, 55). It has been shown that T6SS targeting the peptidoglycan effector Tlde1 inhibits cell division and alters bacterial morphology by interfering with peptidoglycan synthesis (56). Periplasmic expression of VgrG3^cp^ induced a series of events between 0 and 3 h. First, intoxicated cells affected division and formed spherical cells but still continued growth in volume. VgrG3^cp^-mediated toxicity by disrupting peptidoglycan synthesis is consistent with the finding that division septum closure was broken by peptidoglycan hydrolase. Second, we also observed the phenotype that cells were lysed and lost membrane potential simultaneously (Movies S2 and S3), which fit with the idea that the peptidoglycan cross-link rate between NAM and NGM was decreased (56). When the peptidoglycan synthesis rate of a cell lags behind whole-cell growth, the uneven distribution of the peptidoglycan stem alters the integrity of the cell wall, which increases cell sensitivity to osmotic pressure and results in cell death (57).

Environmental strain E1 possesses strong intra- and interspecies antibacterial activities via a T6SS (Fig. S1C and D). Strain E1 encodes membrane-disrupting VasX and potential lipase effector Tle1 in addition to VgrG3^cp^, with the result that E1 wild type has a stronger competition capacity against A. hydrophila than a 2Δ*eff* mutant (only active VgrG3^cp^). Our study demonstrated that VgrG3^cp^ had widespread toxicity for V. cholerae, E. coli, and A. hydrophila. Even though effector proteins target the same substrate, such as acting on cell wall VgrG3 and VgrG3^cp^ encoded by V. cholerae, the specificity of an effector-immunity protein pair determines whether they are incompatible with each other, which has important implications for maintaining ecosystem homeostasis and the genetic diversity of V. cholerae.

## MATERIALS AND METHODS

### Strains and growth conditions.

All bacterial strains and plasmids used in this study are listed in Table S1 in the supplemental material. Strains were grown at 37°C in LB medium with shaking at 200 rpm. When required, antibiotics were added in medium with the following final concentrations: 10 μg/mL chloramphenicol (Cm), 100 μg/mL ampicillin (Amp), 50 μg/mL kanamycin (Km), and 10 μg/mL nalidixic acid (Nal).

The plasmid constructions and bacterial cell transformations were performed following standard molecular cloning procedures. Genetic deletions in V. cholerae were generated by allelic exchange and sucrose counterselection with the suicide vector pDS132 (58). Briefly, the 800-bp upstream fragment and the 800-bp downstream fragment of the target gene were amplified; PCR products were ligated into a pDS132 vector digested with the restriction enzymes XbaI and SacI via a Gibson assembly system. Double-crossover cells were screened on a 10% sucrose LB agar plate without salt. The deletion mutant was confirmed by PCR and sequencing. For expression vectors, the target genes were amplified and ligated into the corresponding vectors digested with the indicated restriction enzymes. All oligonucleotide primers are listed in Table S2.

### Interbacterial competition assays.

For quantitative killing assays, overnight cultures were diluted to an OD_600_ of ~1.0. The mixture was spotted onto LB agar with 0.22-μm polyvinylidene fluoride (PVDF) membrane and incubated at 37°C. After 4 h, surviving cells were enumerated by 10-fold serial dilutions on selective recovery plates.

### Protein secretion assays.

Overnight cultures were inoculated (1:100 dilution) into fresh LB medium supplemented with the appropriate antibiotics and incubated with shaking at 37°C. Cells and supernatant were collected when bacteria had grown to an OD_600_ of ~0.9. If indicated, bacterial strains expressing recombinant plasmid pSRKKm-HA-*vgrG3^cp^* were induced for 2 h by the addition of 30 μM isopropyl-β-d-thiogalactopyranoside (IPTG) at an OD_600_ of ~0.6 and harvested by centrifugation. The supernatant was filtered with a 0.22-μm PVDF filter membrane and precipitated in trichloroacetic acid at a final concentration of 10% at 4°C for at least 30 min. After centrifugation, the concentrated proteins were washed twice with 100% acetone and centrifuged at 4°C. The precipitates from supernatant and whole cells were resuspended in SDS-loading dye and boiled for 10 min before SDS-PAGE analysis.

### Growth inhibition assays.

E. coli BL21(DE3) cells expressing effectors from pET30a or pBAD24 and immunity proteins from pSRKKm (59) were grown in LB supplemented with appropriate antibiotics. Cells were normalized to an OD_600_ of ~1.0 before serial dilution in 0.8% NaCl. Each dilution (5-μL volume) was spotted on LB agar containing the appropriate antibiotics and inducer (50 μM IPTG for pSRKKm and pET30a; 0.2% arabinose for pBAD24) or repressor (0.2% glucose for pBAD24). Plates were grown overnight and imaged. Where stated, the optical density was measured at the indicated times.

### Time-lapse microscopy assays.

Overnight-grown BL21(DE3) cells bearing effectors were inoculated (1:100 dilution) into fresh medium and grown to an OD_600_ of ~1.0. Cells from 1 mL of culture were washed once in 0.5× phosphate-buffered saline (PBS) and concentrated 10 times. One-microliter aliquots of the concentrated cells were subsequently spotted on a thin pad of 1.5% agarose–low-salt LB containing 0.2% arabinose and covered with a glass coverslip. Bacteria were immediately imaged during an observation period of 3 h at 37°C using a Zeiss LSM900 (Carl Zeiss) inverted microscope with a Definite Focus 2.0 system and 100× oil objective lens (Carl Zeiss). Bacterial growth was observed continuously for 3 h, and images was obtained every 30 s. All the microscopy images were processed using ZEN Blue Lite software.

### Peptidoglycan purification and enzymatic assays.

PG was extracted following a previously published protocol (44, 45). Briefly, 2 liters of overnight culture (E. coli MG1655) was pelleted and resuspended in 40 mL 0.025 M potassium phosphate, then added dropwise to an equal volume of boiling 8% SDS. The mixture was boiled for 3 h and placed at room temperature overnight. Then, the mixture was ultracentrifuged at 100,000 × *g* at room temperature to concentrate PG. The pellet was washed with distilled H_2_O (dH_2_O) four times to remove the residual SDS. Precipitates were resuspended with 10 mM Tris-HCl buffer (pH 7.0) and digested with trypsin and DNase I at 37°C overnight. The digested products were further centrifugation at 100,000 × *g* for 1 h and precipitates were resuspended in dH_2_O. Boiling 8% SDS was added and mixtures were boiled for 1 h. After ultracentrifugation, the pellet was washed four times to obtain clear PG samples.

For PG-degrading assays, purified E. coli PG (100 μg) was incubated with 10 μg VgrG3^cp^C and its variant in 100 μL of reaction buffer (20 mM HEPES, pH 6.8) at 37°C, and the OD_600_ was measured at the indicated time points. For analysis of PG-digested products. The purified PG (500 μL) was incubated with 100 μg protein for 12 h. An equal volume of methanol was added into the mixtures. After centrifugation at 12,000 × *g* for 10 min, the supernatant was collected and subjected to analysis by UPLC-QTOF-MS. The Acquity UPLC HSS T3 column was chosen as the solid phase, and solvent A (water with 0.1% [vol/vol] formic acid) and solvent B (methanol) were chosen as the mobile phase. Separation was achieved with a linear gradient of solvent B from 1% to 20% in 65 min, followed by a gradient from 20% to 100% in 1 min and then retained for 5 min, and finally flushed with 99% solvent A for 4 min.

### Protein purification.

The *tsiV3^cp^* and truncated C-terminal *vgrG3^cp^* (VgrG3^cp^C) were cloned into the expressing vector pET30a with a His tag and pGEX-6p with a GST tag, respectively. BL21(DE3) cells with recombinant proteins were grown to an OD_600_ of ~0.8 and induced with addition of IPTG (final concentration, 100 μM) and culturing at 16°C overnight. Cell cultures were harvested by centrifugation and lysed by sonication. Whole-cell lysate was centrifuged at 4°C to remove precipitate, and then the supernatant was loaded on a Ni^2+^-nitrilotriacetic acid (NTA) column or GST Sefinose resin column to purify the proteins of interest. The GST recombinant protein purification kit (catalog number C600327-0001) and Ni-NTA protein purification kit (catalog number C600332-0001) were purchased from Sangon Biotech. All eluted proteins were further purified with the AKTA Explorer protein purification system.

### Western blotting.

Proteins were loaded on an SDS-PAGE gel and run at 160 V, followed by transfer to a polyvinylidene difluoride (PVDF) membrane for 80 min at 270 mA using a Trans-Blot Turbo transfer system. The protein-bound PVDF membrane was blocked with 5% (wt/vol) nonfat milk in Tris-buffered saline with Tween 20 buffer for 1 h at room temperature. The membrane was incubated with primary antibody and secondary antibody and imaged. The primary antibodies (anti-HA and anti-RpoB) and the secondary antibodies (goat anti-mouse IRDye and goat anti-rabbit IRDye) were purchased from Sigma. The Hcp polyclonal antibodies were made by Shanghai Youlong Biotech.

### GST pulldown assay.

A GST pulldown assay was employed to identify the interactions between VgrG3^cp^C and TsiV3^cp^. Briefly, GST-VgrG3^cp^C proteins were incubated with prepared glutathione-Sepharose beads on a rotating incubator for 2 h at 4°C, and then the beads were collected and washed three times with PBS. Purified His-TsiV3^cp^ proteins were added to the beads and incubated for 1 h at 4°C. The beads were washed with wash buffer (20 mM HEPES [pH 7.6], 0.1 M KCl, 1 μM EDTA, 10% [vol/vol] glycerol, 0.02% [vol/vol] NP-40) five times. The bound proteins were analyzed by SDS-PAGE.

### Bioinformatics analysis.

VgrG3^cp^ sequences were analyzed with BLASTp (using the nonredundant protein sequences database) to identify homologs. Protein sequences of VgrG3^cp^ homologs (Data Set S1 in the supplemental material) that included both the *N* terminus of VgrG and pesticin-like domain were downloaded from the NCBI database (60). Homologs were aligned using GeneDoc software, and maximum likelihood phylogeny was performed using MEGA 6.0 with 1,000 bootstraps (61).
