# Supplementary material for: Pesticin-Like Effector VgrG3cp Targeting Peptidoglycan Delivered by the Type VI Secretion System Contributes to Vibrio cholerae Interbacterial Competition
Source: Microbiol Spectr. 2023 Jan 10;11(1):e04267-22. doi: 10.1128/spectrum.04267-22 (PMC9927483; doi:10.1128/spectrum.04267-22)
Supplement: Supplemental file 2 — Fig. S1 to S3, Tables S1 and S2, and legend for Movies S1 to S3. Download spectrum.04267-22-s0002.pdf, PDF file, 1.0 MB [file spectrum.04267-22-s0002.pdf]

Supplementary Information

RT-qPCR analysis.

Overnight culture was transferred with 1:100 dilutions into fresh LB media and grown to OD_{600} ~0.9. Total RNA of bacterial cells was extracted using solution RZ (TIANGEN). Single-stranded cDNA was synthesized from 1 μg of total RNA by using HiScript® III RT SuperMix (Vazyme, R323) with random hexamers as a primer in a 20 μL reaction mixture. The resulting cDNA mixture was diluted to 10 ng/μL as a template for the subsequent quantification assays. Reverse transcription-quantitative PCR (RT-qPCR) was carried out by using the LightCycler® 96 SW (Roche) with specific primers as described in Supplementary Table 2. The relative abundance of *V. cholerae* 16S gene was used as the internal standard to normalize results. The fold change in gene transcription was calculated using the comparative threshold cycle (CT) method(1).

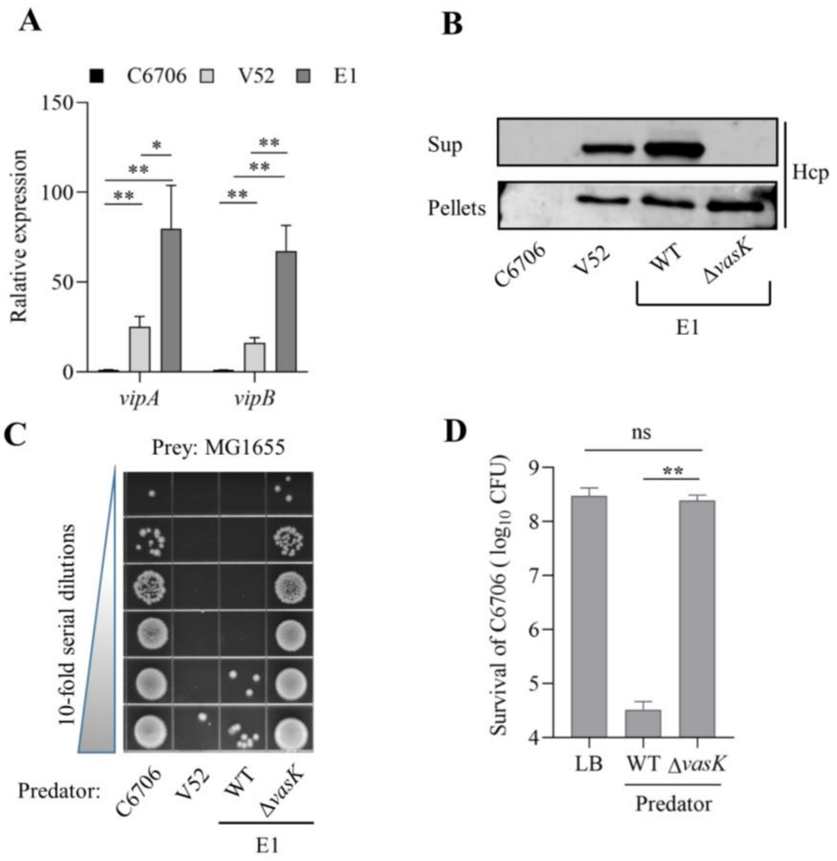

FIG S1 Environmental strain E1 constitutively expresses T6SS. (A) qPCR analysis of T6SS representative genes in *V. cholerae* strains employed. (B) Hcp secretion assays of *V. cholerae*. C6706, V52, E1 Wild type and E1 Δ*vasK* mutant. (C) Competition assays of *V. cholerae* against *E. coli*. Strains in (B) were coincubated with *E. coli* MG1655. Strain C6706 was a negative control and V52 was a positive control. The representative data from three independent biological replicates were displayed. (D) *V. cholerae* intra-species competition assay. E1 strains (WT, Δ*vasK*) were coincubated with prey C6706. Error bars (A and D) represent the mean ± SD of three independent biological replicates. Significance was calculated using unpaired student's *t* test, ns, non-significant;* $P < 0.05$； ** $P < 0.01$.

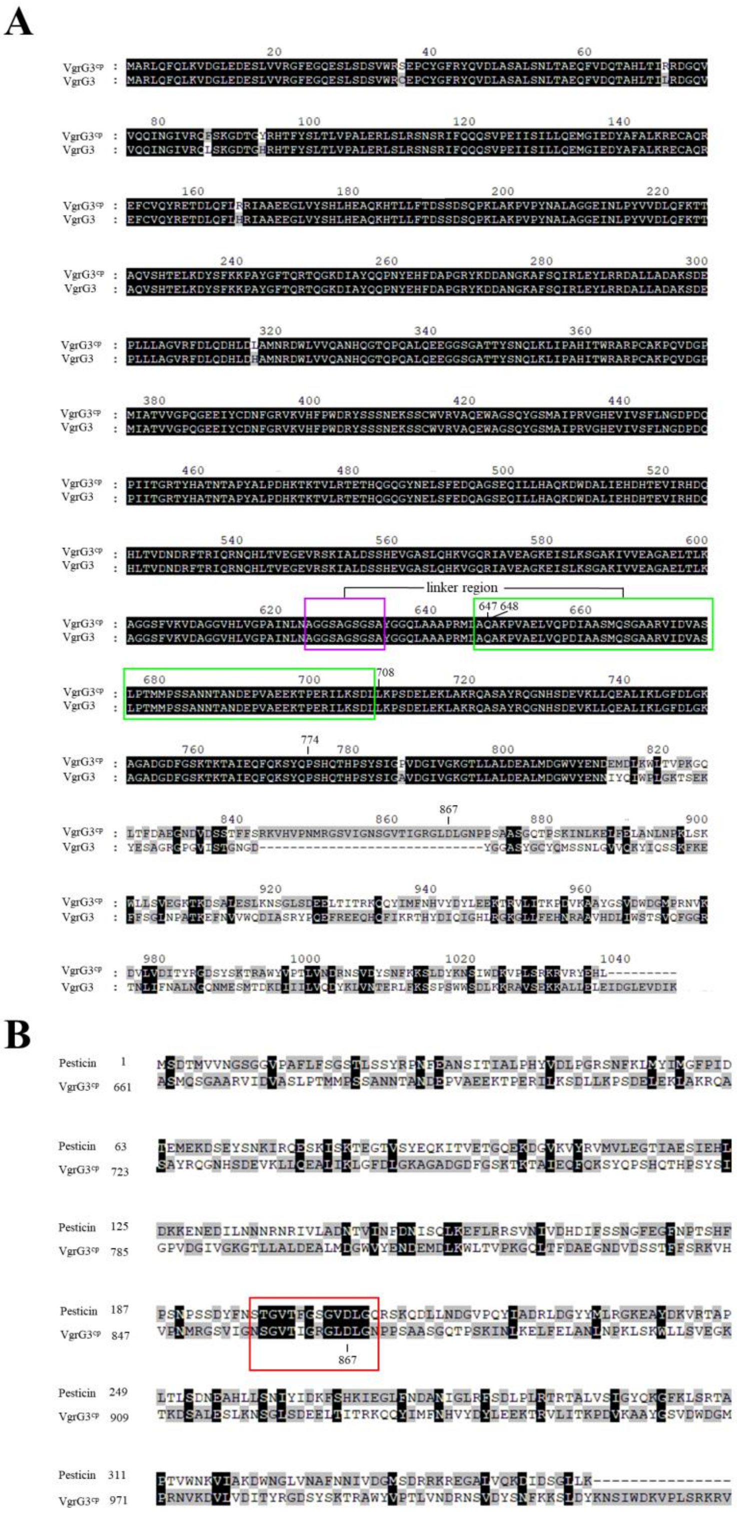

FIG S2 Comparison of amino acid sequences of VgrG3^{cp} and VgrG3 (accession number: WP_000113295). The conservative residues are indicated in black background, the

residues marked in violet box are the flexible linker sequence, and the residues highlighted in green box contain a canonical signal peptidase cleavage motif (A). Analysis of conserved domain between VgrG3^{cp}C and pesticin (accession number: WP_002218509). The catalytic core region of pesticin is highlighted in red box (B).

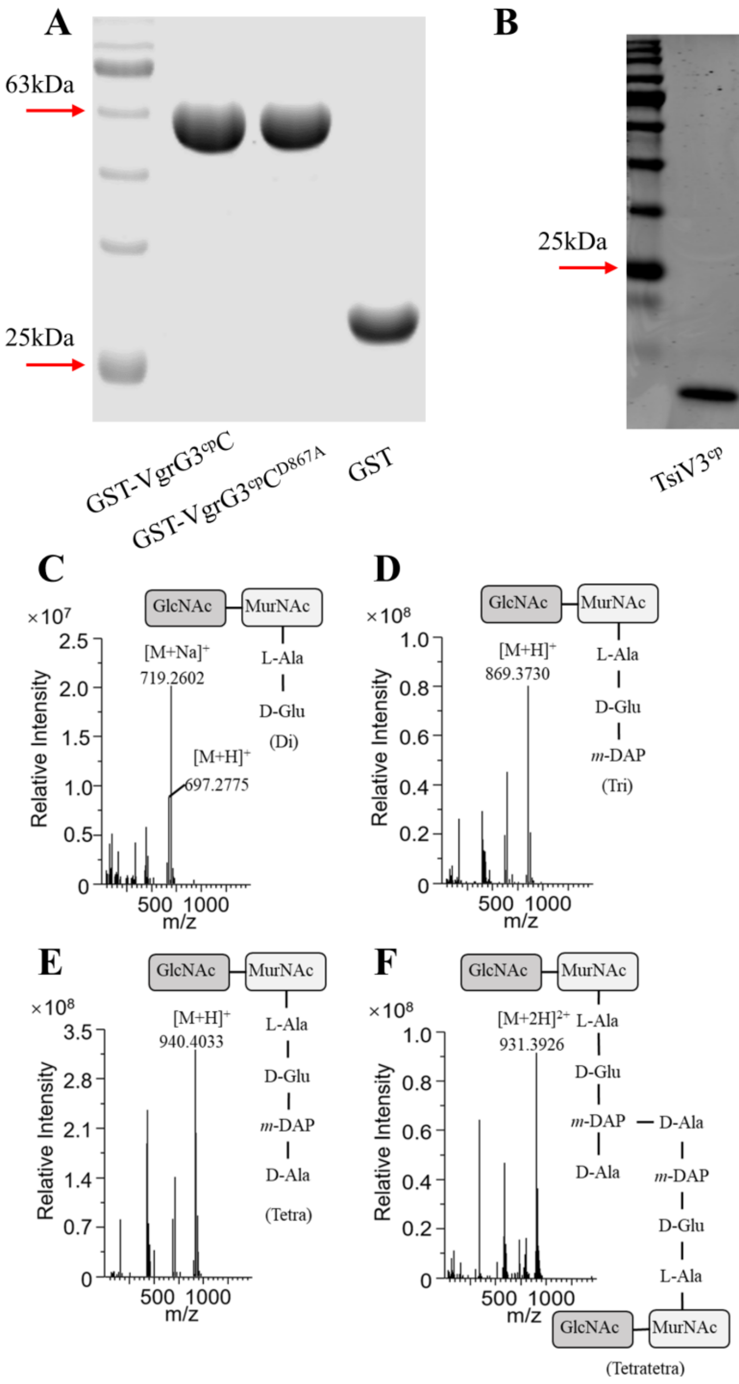

FIG S3 (A and B) Purification of the truncated VgrG3^{cp} and its cognate protein TsiV3^{cp}. **(**C to F) The analysis of PG-digestion products by VgrG3^{cp}C and its catalytic mutant. Identified NAG (N-acetylglucosamine)-NAM (N-acetylmuramic acid) products are indicated. C, D, E and F represent Di, Tri, Tetra and Tetratetra, respectively.

Movie S1, S2 and S3. Time-lapse microscopy of *E. coli* BL21 cells expressing TAT-VgrG3^{cp}C^{D867A} (S1) TAT-VgrG3^{cp}C (S2 and S3) or grown on LB agar pads containing 0.2% L-arabinose and Green fluorescent dye DiBAC4(3). Movie S3 was used to highlight the result of VgrG3^{cp}-induced representative phenotype. Spherical cells and burst cells were indicated with yellow arrow and green arrow, respectively. Scale bar, 5 μm. Related to Figure 2A-D. Total duration was at least 3 h, recorded in 30 s intervals.

Table 1 strains and plasmids

**Strain**	Genotype	Description	Source
V. cholerae	C6706	Toxigenic strain, O1 serogroup	Lab stock
	E1	Environmental strain, O1 serogroup, no TCP and CT, parental strain	Lab stock
	Δ*vasK*	*vasK* gene deletion mutant from E1	This study
	$\Delta vgrG3^{cp}/tsiV3^{cp}$	$vgrG3^{cp}$ *and* $tsiV3^{cp}$ gene deletion mutant from E1	This study
	$\Delta vgrG3^{cp}$	$vgrG3^{cp}$ gene deletion mutant from E1	This study
	$\Delta vgrG3^{cp}+C$	$\Delta vgrG3^{cp}$ with the vector pBAD24-$vgrG3^{cp}$	This study
E. coli	BL21(DE3)	Host bacteria of protein expression and purification	Lab stock
	DH5α	Construction of recombination plasmid	Lab stock
	BW20676	For conjunction assays	Lab stock
	MG1655	Used to extract Peptidoglycan and competition assay	Lab stock
A. hydrophila	BJ017		(2)
	BJ018	Clinical strain	
	BJ054		

**Plasmid**	Description	Reference
pBAD24	Arabinose-induced expression vector, Amp^R	(3)
pDS132	Suicidal conjugation vector for chromosomal allelic changes Cm^R	(4)
pSRKKm	IPTG-induced expression vector for complementation assays, Km^R	(5)
pGEX6P	IPTG-induced expression vector with GST tag for protein purification, Amp^R	Lab stock

pETM3C	IPTG-induced expression vector with 6* his tag for protein purification, Amp^{R}	Lab stock
pET30a	IPTG-induced expression vector with 6* his tag for gene complementation, Km^{R}	Lab stock
pBAD24-*tat-vgrG3*^{cp}	For expressing VgrG3^{cp} in the periplasm, Arabinose inducible	This study
pSRKKm-*tsiV3*^{cp}	For expressing TsiV3^{cp}, IPTG inducible	This study
pGEX6P-*vgrG3*^{cp}*C*	For purifying VgrG3^{cp}C, IPTG inducible	This study
pETM3C-*tsiV3*^{cp}	For purifying TsiV3^{cp}, IPTG inducible	This study
pSRKKm-*ha-vgrG3*^{cp}	For overexpressing HA-VgrG3^{cp}, IPTG inducible	This study
pET30a-*vgrG3*^{cp}	pET30a carrying the *vgrG3*^{cp} full length, Km^{R}	This study
pET30a-*vgrG3*^{cp}*N*	pET30a carrying the *vgrG3*^{cp} 1-2430, Km^{R}	This study
pET30a-*vgrG3*^{cp647}	pET30a carrying the *vgrG3*^{cp} 1939-end, Km^{R}	This study
pET30a-*vgrG3*^{cp648}	pET30a carrying the *vgrG3*^{cp} 1942-end, Km^{R}	This study
pET30a-*vgrG3*^{cp}*C*	pET30a carrying the *vgrG3*^{cp} 2122-end, Km^{R}	This study
pET30a-*vgrG3*^{cp774}	pET30a carrying the *vgrG3*^{cp} 2320-end, Km^{R}	This study
pDS132-*vgrG3*^{cp}	Recombinant vector to construct chromosomal clean deletion of *vgrG3*^{cp}	This study
pDS132-*vgrG3*^{cp}/*tsiV3*^{cp}	Recombinant vector to construct chromosomal clean deletion of *vgrG3*^{cp}/*tsiV3*^{cp}	This study

Table 2 Primers used in this study.

Primer name	Sequence
pET30a- *vgrG3*^{cp}-F	GGAATTCATGGCAAGGTTACAGTTTCA
pET30a- *vgrG3*^{cp}-R	CCCAAGCTTTTAAAGATGTTCATATCTTACTCTC

pET30a- *vgrG3*^{cp}*N*-R	CCCAAGCTTTTATTCATACACCCACCCATCCA
pET30a- *vgrG3*^{cp647}-F	GGAATTCATGCAAGCTAAACCAGTAGCTGA
pET30a- *vgrG3*^{cp648}-F	GGAATTCATGGCTAAACCAGTAGCTGAATTG
pET30a- *vgrG3*^{cp}*C*-F	GGAATTCATGCTCAAACCATCCGATGAGTT
pET30a- *vgrG3*^{cp774}-F	GGAATTCATGCCAAGCCACCAAACACACCC
pDS132- *vgrG3*^{cp}-1	GGGTTAAAAAGGATCGCTTCTAGAACTCTAGGTGTACTTGGG
pDS132- *vgrG3*^{cp}-2	TTCATATCTTACCTGTAACCTTGCCATGCT
pDS132- *vgrG3*^{cp}-3	CAAGGTTACAGGTAAGATATGAACATCTTTAAAAAAAT
pDS132- *vgrG3*^{cp}-4	ATTCCCGGGAGAGCTCGACTTCGCCAAACAC
pDS132-*vgrG3*^{cp}*/tsiV3*^{cp}-1	TGATGGGTTAAAAAGGATCGCTTCTAGACTAGTCGGTCTAAGCAC
pDS132- *vgrG3*^{cp}*/tsiV3*^{cp}-2	GCGGACTGGCTTTCTACGTGCTTGGCGAGTAACTTAG
pDS132- *vgrG3*^{cp}*/tsiV3*^{cp}-3	AAATTAGCATGAGTAGCACATCAGAAGAACTCGTCAAG
pDS132- *vgrG3*^{cp}*/tsiV3*^{cp}-4	CAATTTGTGGAATTCCCGGGAGAGCTCCTCATGATTCAGCAGTG
pBAD24-*tat-1*	TGGGCTAGCAAGAGGAATTCATGAACAATAACGATCTCTTT
pBAD24-*tat- vgrG3*^{cp}*C*-2	GGTTTGAGCGCCGCTTGCGC
pBAD24-*tat-vgrG3*^{cp}*C*-3	AGCGGCGCTCAAACCATCCGATGA
pBAD24-*tat*-4	CCGCCAAAACAGCCAAGCTTTTAAAGATGTTCATATCTTACTCTC
pBAD24-*tat-vgrG3*^{cp774}-2	TGGCTTGGCGCCGCTTGCGCCGCAGTC
pBAD24-*tat-vgrG3*^{cp774}-3	AGCGGCGCCAAGCCACCAAAC
pDS132--*vgrG3*^{cpD867A}-1	AAGGATCGCTTCTAGATCGATGCAGGTGGCGTGCAC

pDS132-*vgrG3*^{cpD867A}-2	GGATTACCTAAAGCAAGTCCCCGCCCGATGGT
pDS132-*vgrG3*^{cpD867A}-3	GGCGGGGACTTGCTTTAGGTAATCCTCCAAGT
pDS132-*vgrG3*^{cpD867A}-4	ATTCCCGGGAGAGCTCTTAAAGATGTTCATATCTTACTCTC
pSRKKm- *tsiV3*^{cp} -F	GGGGGATCCACTAGTTCTAGATTATTCAGCCTTATCGCATT
pSRKKm- *tsiV3*^{cp} -R	CACACAGGAAACAGCATATGAACATCTTTAAAAAAATCATATAT
pGEX6P-*vgrG3*^{cp}*C*-F	CGGGATCCCTCAAACCATCCGATGAGTTAG
pGEX6P-*vgrG3*^{cp}*C*-R	CGGAATTCTTAAAGATGTTCATATCTTACTCTC
pETM3C- *tsiV3*^{cp} -F	AGGGGCCCGGATCCGAATTCGTTGGGGGTAGTATTCCTTC
pETM3C- *tsiV3*^{cp} -R	TCGAGTGCGGCCGCAAGCTTTTATTCAGCCTTATCGCATTT
pSRKKm-*ha*-*vgrG3*^{cp}-1	CGCGGATCCATGGCAAGGTTACAGTTTC
pSRKKm-*ha*-*vgrG3*^{cp}-2	CCCAAGCTTTTAAGCGTAGTCTGGGA
q-*vipA*-F	CATTGGAAGAGCGTGCAAC
q-*vipA*-R	GCATTCTCATCATCAGTCAGC
q-*vipB*-F	TGCTAAATATCGCTGGTGTC
q-*vipB*-R	GCTTTGCAATGCACCCATAG

REFERENCES

1. Livak KJ, Schmittgen TD. 2001. Analysis of relative gene expression data using real-time quantitative PCR and the 2(-Delta Delta C(T)) Method. Methods 25:402-8.

2. Zhou Y, Yu L, Nan Z, Zhang P, Kan B, Yan D, Su J. 2019. Taxonomy, virulence genes and antimicrobial resistance of *Aeromonas* isolated from extra-intestinal and intestinal infections. BMC Infect Dis 19:158.

3. Guzman LM, Belin D, Carson MJ, Beckwith J. 1995. Tight regulation, modulation, and high-level expression by vectors containing the arabinose PBAD promoter. J Bacteriol 177:4121-30.

4. Philippe N, Alcaraz JP, Coursange E, Geiselmann J, Schneider D. 2004. Improvement of pCVD442, a suicide plasmid for gene allele exchange in bacteria. Plasmid 51:246-55.

5. Khan SR, Gaines J, Roop RM, 2nd, Farrand SK. 2008. Broad-host-range expression vectors with tightly regulated promoters and their use to examine the influence of TraR and TraM expression on Ti plasmid quorum sensing. Appl Environ Microbiol 74:5053-62.
